# Highly Emissive Colloidal Nanocrystals of a “2.5-Dimensional” Monomethylhydrazinium Lead Bromide

**DOI:** 10.1021/jacs.4c16698

**Published:** 2025-02-12

**Authors:** Viktoriia Morad, Taehee Kim, Sebastian Sabisch, Simon C. Boehme, Simone Delessert, Nadine J. Schrenker, Sara Bals, Gabriele Rainò, Maksym V. Kovalenko

**Affiliations:** † Laboratory of Inorganic Chemistry, Department of Chemistry and Applied Biosciences, ETH Zürich, Zürich 8093, Switzerland; ‡ Laboratory for Thin Films and Photovoltaics, Empa − Swiss Federal Laboratories for Materials Science and Technology, Dübendorf 8600, Switzerland; § Electron Microscopy for Materials Science (EMAT) and NANOlab Center of Excellence, University of Antwerp, Antwerp 2020, Belgium

## Abstract

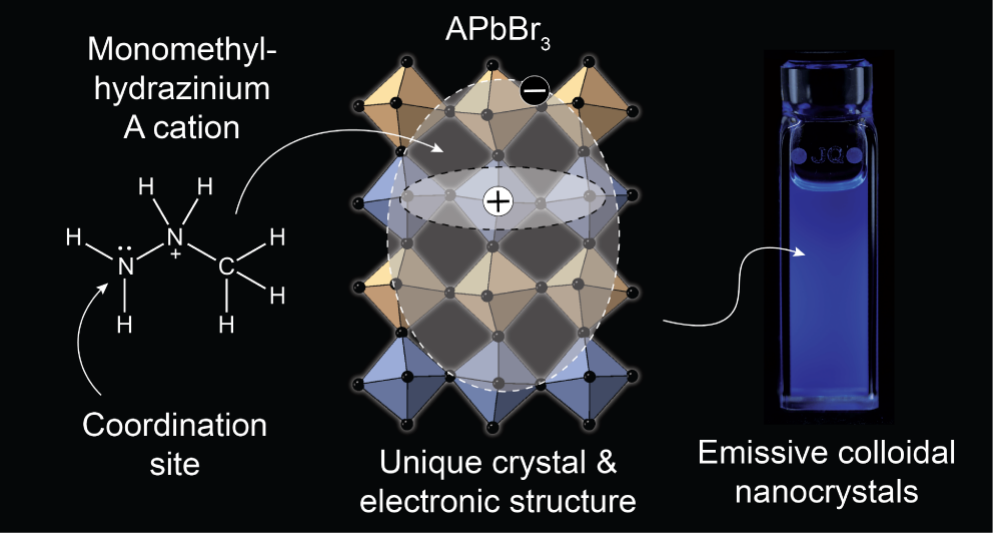

The ability to control materials at the nanoscale has advanced optoelectronic devices, such as LEDs, displays, and quantum light sources. A new frontier is controlling exciton properties beyond quantum size confinement, achieved through single monolayer heterostructures. In the prototypical example of transition metal dichalcogenide heterostructures and moiré superlattices, excitons with long lifetimes, strong binding energies, and tunable dipole moments have been demonstrated and are ideal for optoelectronics and quantum applications. Expanding this material platform is crucial for further progress. This study introduces colloidal nanocrystals (NCs) of monomethylhydrazinium lead bromide (MMHPbBr_3_), a novel lead halide perovskite (LHP) with a unique “2.5-dimensional” electronic structure. While the spatial dimensionality of the NC extends in all three dimensions, these NCs exhibit excitonic properties intermediate between 2D and 3D LHPs. Density functional theory (DFT) calculations show that MMHPbBr_3_ features spatially separated electron and hole wave functions, with electrons delocalized in 3D and holes confined in 2D monolayers. Synthesized via a rapid colloidal method, these NCs were characterized by using techniques such as 4D-STEM and nuclear magnetic resonance, confirming their monoclinic structure. Optical analysis revealed size-dependent properties and 3D quantum confinement effects, with three distinct photoluminescence (PL) bands at cryogenic temperatures corresponding to excitons with varying interlayer coupling. PL spectroscopy of single MMHPbBr_3_ NCs reveals their photon emission statistics, expanding their potential for unconventional quantum material designs.

## Introduction

**Figure 1:**
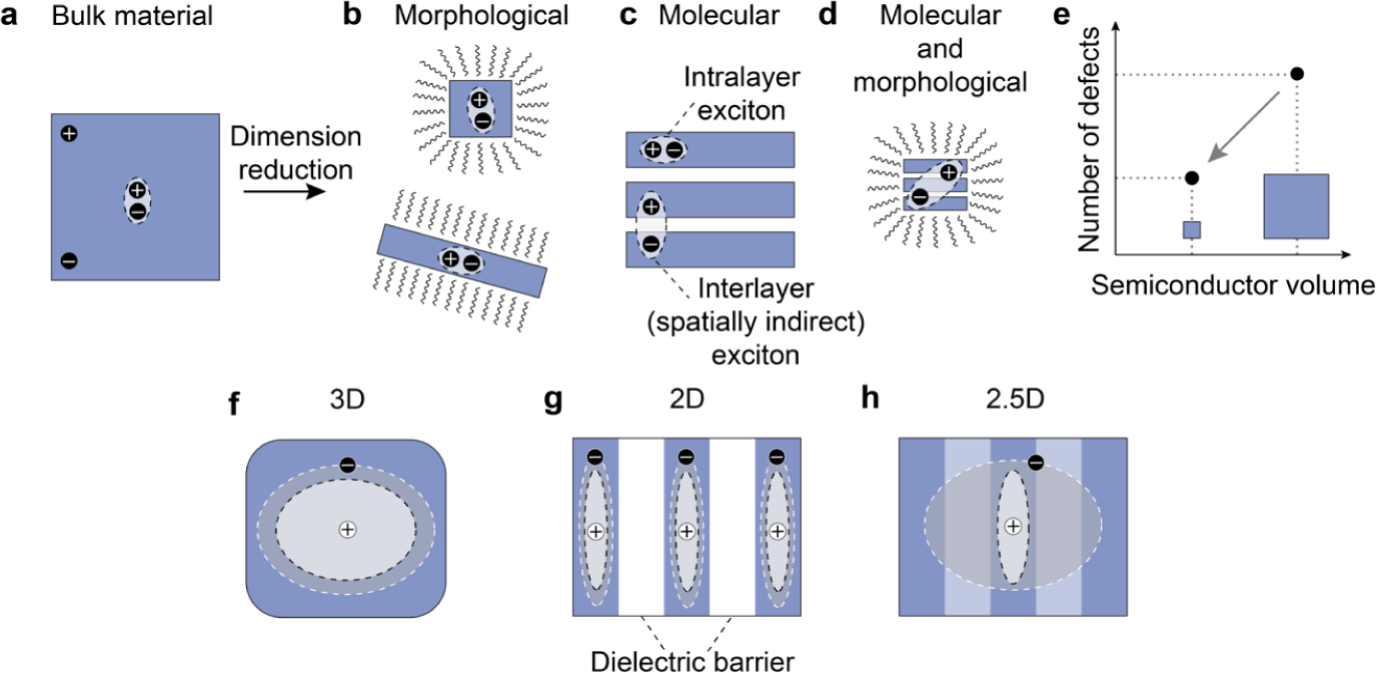
(a–d) Various examples of dimensional reduction of a bulk (a) semiconductor to achieve exciton confinement: morphological (b), molecular (c), and combined molecular and morphological (d). (e) The number of defects in a semiconductor decreases with the number of atoms (volume) and nanostructuring offers a pathway to decrease the number of defects per exciton. (f–h) Examples of confinement potentials: 3D confinement in the whole volume of the material (f), an ordered periodic 2D potential in a layered material (g), and a mixed case, where one of the carriers delocalized in a 2D space and the other in a 3D space (2.5D) (h).

Materials with spatially confined and periodic electronic structures, for example, atomically thin or layered semiconductors and their heterostructures, have recently emerged as promising platforms to engineer electron–hole pairs (excitons) bound by Coulomb forces.^1^ Two common dimensionality reduction strategies are employed to achieve excitonic quantum and dielectric confinement in semiconductors (Figure 1a): (i) morphological reduction via nanostructuring of bulk materials (Figure 1b), or (ii) atomic-scale dimensionality reduction via ordered modulation of the bulk crystal structure (Figure 1c). Layered multi-quantum-well heterostructures have the advantage of small interlayer distances, offering a path for precise control over the exciton internal spatial distribution, such as separating electrons and holes into different layers, which can alter the binding energy, lifetime, mobility, and coherence time of the resulting interlayer exciton – all properties attractive for quantum light technologies.^2^

Combining the two strategies and making stable colloidal nanostructures out of a layered material (Figure 1d) is a formidable scientific challenge: layers are often held together by weak van der Waals forces that cannot compete with ligand binding, leading to uncontrolled exfoliation into nanosheets. Yet, the allure of a semiconductor in a colloidal nanoform is unmatched. Aside from facile ink preparation and quantum confinement effects, simply scaling down the volume of the material per se reduces the total number of defects (Figure 1e) that negatively affect exciton dynamics.

Lead halide perovskite (LHP) semiconductors exhibit remarkable structural tunability, offering a rich compositional and morphological variety suited for diverse cutting-edge optoelectronic applications.^3^ For example, 3D confinement is characterized by electron and hole wave functions delocalized over the entire volume of the material (Figure 1f) and is represented by quantum dots of LHPs. In contrast, 2D confinement means that electrons and holes are localized in-plane, creating intralayer excitons (Figure 1g), with numerous layered perovskite phases.^4^ The only example of stable colloidal nanocrystals (NCs) produced from a layered material is the fully inorganic Ruddlesden–Popper Cs_2_PbCl_2_I_2_ phase, where ionic forces hold the layers together, evidencing that structurally rigid materials are easier to formulate into robust NCs.^5^

To achieve fine control over exciton properties, it is beneficial to spatially separate electrons and holes, for example, by having a layered structure where the spatial extension of the carriers’ wave-function can have different dimensionality: we call such a case here a 2.5D confinement (Figure 1h). Thus far, correlated excitonic states in LHP materials have been investigated in either 3D-confined structures (superfluorescence in quantum dots) or 2D-confined layers (moiré superlattices).^6^ Examples of hybrid LHP structures with spatially separated electrons and holes have, to date, only been reported under an electric field in the observation of the quantum-confined Stark effect.^7^ Another related example is Ag-doping in Cs_3_Bi_2_Br_9_, which results in the formation of a bound interlayer exciton.^8^ We set out to find an LHP material that can intrinsically host electron and hole with spatial separation in an ordered periodic manner and can be formulated into colloidal NCs.

**Figure 2:**
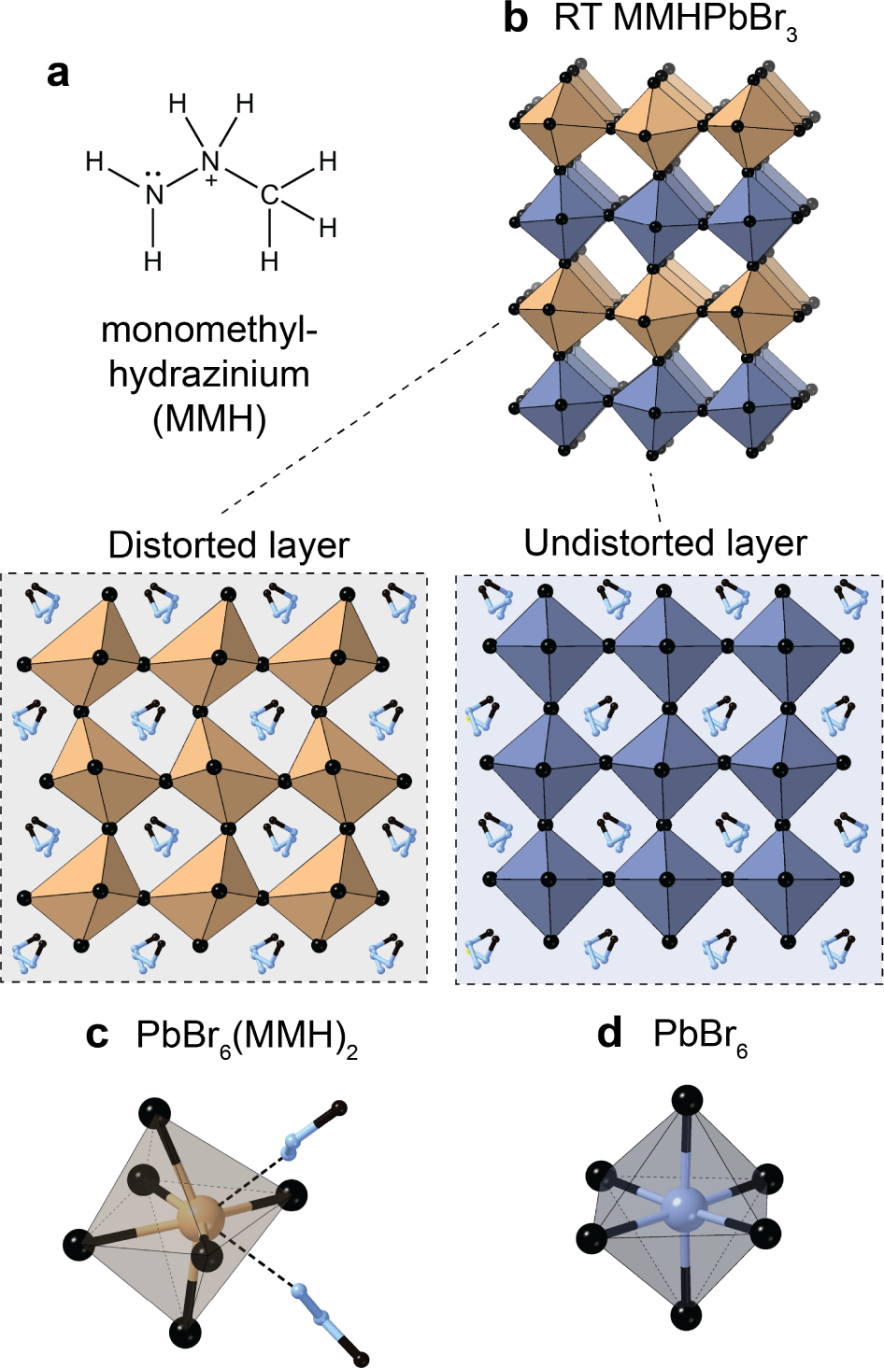
(a) Monomethylhydrazinium (MMH^+^) cation. MMHPbBr_3_ has a 3D perovskite lattice (b) with two distinct alternating layers: one distorted layer (depicted in gold), where PbBr_6_ octahedra are distorted due to coordination of two MMH^+^ cations (c), and one undistorted layer (depicted in blue) comprising undistorted PbBr_6_ octahedra (d).

An archetypical LHP has an ABX_3_ formula, where A is a small cation, either inorganic (Cs^+^) or organic (methylammonium, MA^+^ or formamidinium, FA^+^), B = Pb, Sn, and X = Cl, Br, I. Since the properties of LHPs depend on the composition, every constituent of the perovskite lattice has been scrutinized, and numerous organic cations have been considered for the A-site position.^9^ The rule for retaining a three-dimensional (3D) perovskite lattice is summarized by the Goldschmidt tolerance factor, limiting the size of the A-cation to not much larger than 260 pm. At the edge of the 3D perovskite stability window are a couple of nontrivial organic cations, e.g., monomethylhydrazinium (MMH^+^, 263 pm), azetidinium (AZT^+^, 250 pm), and aziridinium (AZR^+^, 227 pm). While AZR^+^ still preserves a cubic perovskite lattice,^10^ AZT^+^ and MMH^+^ introduce lattice distortions, resulting in the retention of a corner-sharing 3D perovskite motif, albeit with the adoption of a symmetry lower than cubic.^11^ Among other organic cations, MMH^+^ uniquely stands out with its lone electron pair residing on the terminal nitrogen atom (Figures 2a and S1). As a donor, MMH^+^ readily coordinates to metals in many known MMH-metal halide salts.^12^ Recently discovered bulk MMHPbBr_3_ is not an exception.^11b^ It features MMH^+^ cations residing in the A-site of the perovskite structure and simultaneously coordinating with the nitrogen lone pair to one Pb atom together with the perovskite octahedral environment of Br atoms (Figure 2c). The other crystallographically inequivalent Pb atom resides in a conventional octahedral perovskite environment (Figure 2d). Both PbBr_6_ and PbBr_6_(MMH)_2_ octahedron units assemble via corner sharing into a 3D perovskite structure comprising alternating undistorted and distorted layers (Figure 2b). The structure of MMHPbCl_3_ is analogous,^13^ while MMHPbI_3_ has been reported in a 2D layered structure instead.^14^ Moreover, coordination of MMH^+^ to Pb in all related 2D layered or nonperovskite halide phases causes static breaking of symmetry, resulting in nonlinear properties.^13,15^ Such a unique set of features triggers a deeper investigation into the MMHPbBr_3_ photophysical and excitonic properties.

Alternating conducting layers and insulating interlayers is one prominent approach represented by various 2D LHPs, where inorganic perovskite fragments of defined thicknesses are separated by interlayers of organic molecules.^16^ The exploration of the interlayers beyond organic molecules is of great interest, but examples of fully inorganic layered perovskites are scarce.^17^ Despite featuring alternating layers, the unique MMHPbX_3_ structure cannot be outright categorized as one of the two classical electronical types: a 3D perovskite or a layered 2D perovskite with *n* = 1. The bulk material of MMHPbBr_3_ is yellow-colored (optical band gap of 2.54 eV, Figure S2), distinct from all its other APbBr_3_ counterparts, which are orange and have lower band gaps.

The band gap in LHP semiconductors depends on the efficiency of the orbital overlap between adjacent PbBr_6_ octahedra, and the deviation of the Pb–Br–Pb angle from 180° is understood to decrease orbital overlap and can lead to an increased band gap.^8−10a,10b,10c,18a,18b^ The undistorted PbBr_6_ layer (let alone the distorted PbBr_6_(MMH)_2_ layer) features a deviation from the ideal Pb–Br–Pb bond angles (Figure S3). However, such a strong band-gap opening as in MMHPbBr_3_ can also stem from the alternating layered structure, featuring highly bound excitons delocalized in a 2D layer, in analogy to a 2D LHP.

With numerous possible explanations for the observed band gap of MMHPbBr_3_, chemical intuition does not immediately offer a clear picture. The degree of wave-function hybridization between the distorted and undistorted layers, as well as studies of exciton dynamics, can help define where MMHPbBr_3_ falls in its electronic structure classification. In this work, to comprehensively assess the nature of MMHPbBr_3_, we start by analyzing its electronic structure using density functional theory (DFT). Since the excitonic properties of semiconductor materials manifest in the regime of quantum confinement, we leverage room-temperature (RT) synthetic approaches and novel surface-ligand chemistry to produce MHAPbBr_3_ NCs and study their RT and temperature-dependent properties at the ensemble and single-NC level, revealing photophysical processes stemming from their unique crystal structure.

## Results and Discussion

**Figure 3:**
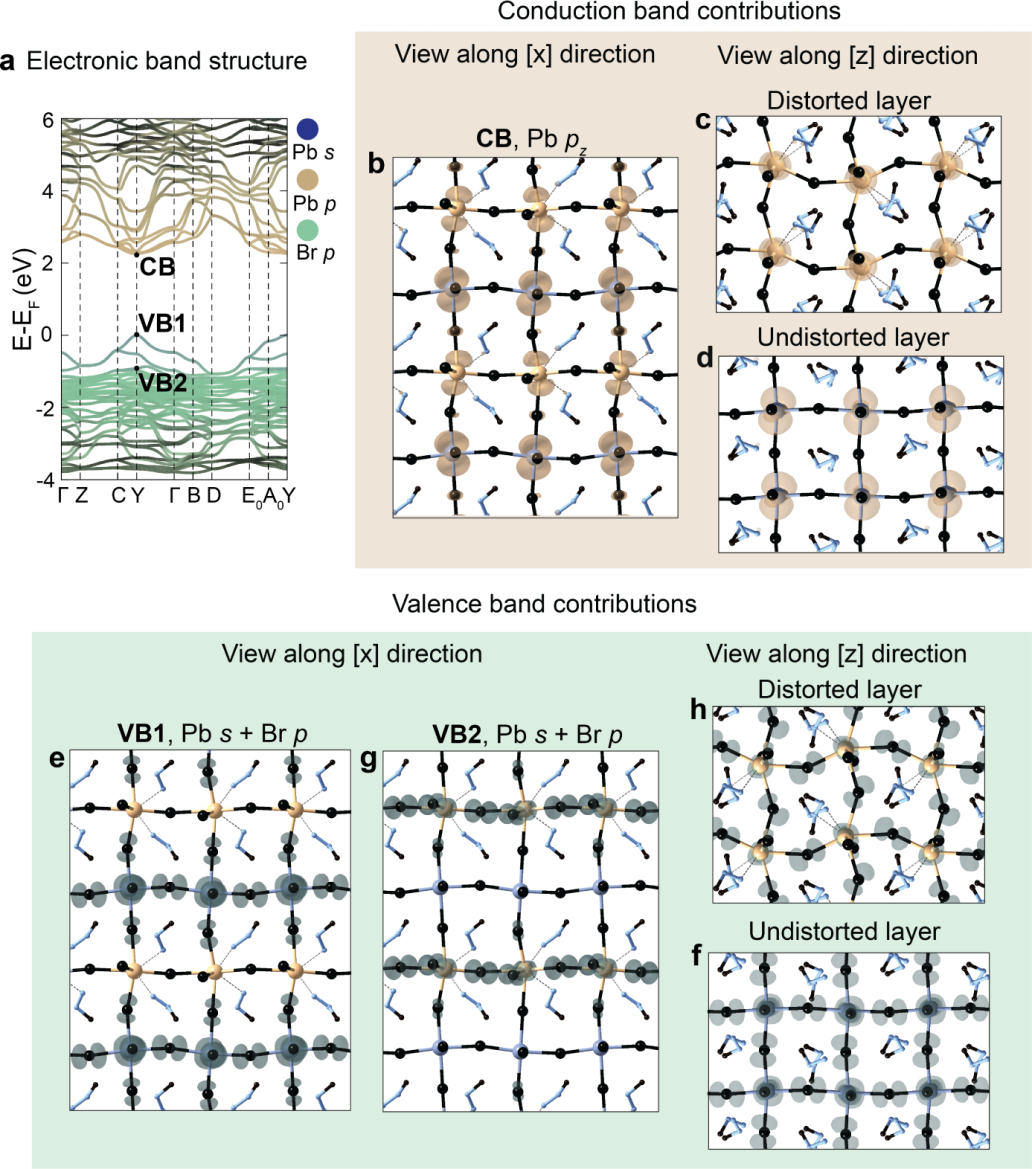
(a) Electronic band structure of bulk MMHPbBr_3_ color-coded with Pb-s, Pb-p, and Br-p contributions. (b–d) Projected atomic orbitals at the CB of MMHPbBr_3_ showing a three-dimensional electron wave function delocalization. (e,f) Projected atomic orbitals at VB1 (Y point and *E* – *E*_F_ = 0 eV) showing hole-wave function delocalization only in the plane of the undistorted layer. (g,h) Projected atomic orbitals at VB2 showing hole-wave function delocalization in the plane of the distorted layer.

To access the electronic structure of MMHPbBr_3_, we analyze its bulk band structure calculated at the DFT level of theory (Figure 3a, see the Methods section in Supporting Information for calculation details). Typically for LHPs, the maximum of the valence band (VB) is composed of Pb s-states and Br p-states, while the minimum of the conduction band (CB) features mostly Pb p-states and some contribution from Br p-states. Atomic projections of the molecular orbitals at the VB maximum and CB minimum represent hole and electron wave functions, respectively, so that the dimensionality and extent of these states can be effectively visualized. The electron wave function in bulk MMHPbBr_3_ is delocalized throughout the whole 3D structure, with contributions from both undistorted and distorted layers (Figure 3b–d). On the contrary, the hole wave function is delocalized only throughout the 2D plane of the undistorted PbBr_6_ layer (Figure 3e,f, VB1). The Pb s- and Br p-hybridized states are localized in the 2D plane of the distorted PbBr_6_(MMH)_2_ layer, deeper in the VB (Figure 3g,h, VB2). These results suggest that MMHPbBr_3_ can theoretically possess carriers with intrinsically different confinement: an electron delocalized in 3D and a hole in 2D, respectively, a property encountered in neither 3D nor 2D layered LHPs. An analogous feature of differing dimensionality in electron and hole wave functions has been observed for the Na-alloyed Cs_2_AgInCl_6_ double perovskite, with the electron delocalized over the 3D structure and the hole confined to a single AgCl_6_ octahedron.^19^ This fascinating electronic structure prompted us to also experimentally study the photophysics of such peculiar 2.5D excitons in MMHPbBr_3_, particularly in the form of colloidal NCs.

**Figure 4:**
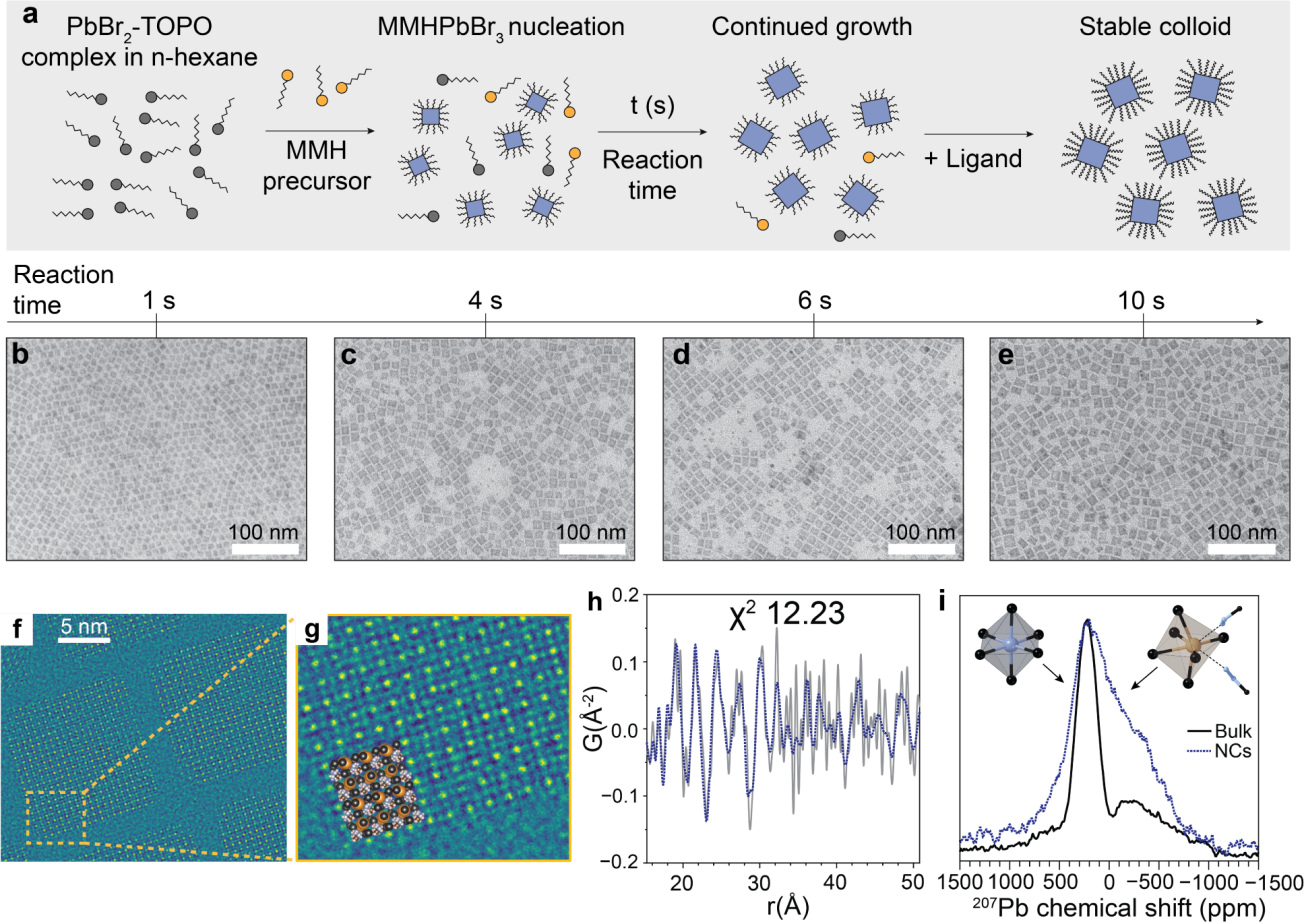
(a) Room-temperature synthesis of MMHPbBr_3_ NCs. (b-e) Size evolution of MMHPbBr_3_ NCs with increasing reaction time. (f) Phase contrast reconstruction from 4D-STEM of the MMHPbBr_3_ NCs, with (g) depicting a magnified region of (f) to show the atomic structure in high resolution and with the model of the structure projected along the [001] crystallographic direction as an overlay (Pb = orange, Br = black, and MMH = blue/white). (h) PDF analysis of the XRD data of MMHPbBr_3_ NCs fitted to the RT crystallographic phase. (i) Solid-state ^207^Pb NMR of the bulk (black solid line) and NC powder (blue dotted line) of MMHPbBr_3_ shows two signals from two different coordination environments of Pb in MMHPbBr_3_.

In the footsteps of previous studies reporting the synthesis of bulk forms of MMHPbBr_3_,^11b^ we report the synthesis of MMHPbBr_3_ in the form of colloidal NCs. Such nanocrystalline forms of this material uniquely enable us to study the emission properties of these unusual 2.5D excitons: while bulk MMHPbBr_3_ is essentially nonemissive, the combination of reduced spatial extent of the NCs and simultaneously ensuring good electronic passivation of the NC surface proves to be an effective route to boost the PL quantum yields (QYs), similarly exploited in many other colloidal semiconductor NCs. To obtain colloidal MMHPbBr_3_ NCs, we adopt a modified RT synthesis procedure,^20^ accounting for the volatility and toxicity of MMH, and minimizing MMH losses during the NC synthesis (see Methods, Table S1, and Figure S4).^20^ Briefly, PbBr_2_ is solubilized in an apolar mixture of *n*-octane and *n*-hexane with tri-*n*-octylphosphine oxide (TOPO). MMH^+^ salt of a long-chain acid is swiftly injected into the Pb precursor, followed by nucleation and growth of MMHPbBr_3_ NCs (Figure 4a). The NC growth can be stopped with suitable capping ligands, like oleylammonium bromide or phosphoalkylamine zwitterions,^21^ since both TOPO and long-chain acids (a mixture of oleic and diisooctylphosphinic acids, OA and DOPA) present in the synthesis are poor capping ligands that do not render long-term colloidal stability and can only sustain NC growth. Commonly for organic–inorganic LHPs, this reaction is fast, but still, the size of the resulting NCs can be tuned with the reaction time (Figure 4b–e). It must be noted that compared to other A-cations (Cs, FA, and MA), the analogous MMHPbBr_3_ PbBr_2_-TOPO and A-DOPA reaction is the fastest under the same conditions, taking only a couple of seconds for NCs to reach a 10 nm size. Among the capping ligands that can stop MMHPbBr_3_ NCs growth – oleylammonium bromide (OAmBr), phosphoethanolammonium (PEA), or phosphopropanolammonium (PPA) – only the latter two zwitterions provide colloidal stability and integrity during NC purification, albeit limited to only one or two cycles (Figure S5). The above observations, both in reaction kinetics and in ligand binding, indicate that the formation of bulk MMHPbBr_3_ is a competing and energetically more favored process, a likely consequence of MMH coordination to Pb and covalent linkage of anionic and cationic sublattices, another unique feature of MMH perovskites. Following synthesis, MMHPbBr_3_ NCs are most conveniently capped by zwitterionic PEA ligands with a branched 2-octyldodecyl aliphatic tail. PEA ligands are sufficiently tightly bound (Figure S6) and allow MMHPbBr_3_ NCs to be purified once and subjected to diverse characterization without compromising their integrity in concentrated and diluted colloids, films, and as NC powders.

Bulk MMHPbBr_3_ is reported to adopt two structural modifications, RT monoclinic and high-temperature (HT) cubic.^11b^ The cubic symmetry of the HT modification is a consequence of the Pb–N bond breaking and the averaged rotation of the MMH cation. To identify which phase is adopted at RT by NCs, we employ a range of analytical techniques. For example, high-resolution 4D-STEM is a useful technique for beam-sensitive materials, like LHP NCs, which are prone to degradation upon exposure to high electron doses.^22^ Phase contrast reconstructions from 4D-STEM of MMHPbBr_3_ NCs reveal good crystallinity (Figure 4f,g) of the synthesized NCs, and the FFT pattern agrees with the [001] zone axis of the monoclinic MMHPbBr_3_ RT phase (Figure S7). Since the 4D-STEM reconstruction contains phase contrast information,^22b^ it enables us to simultaneously image atomic columns of heavy Pb and the light organic MMH cations. Furthermore, we analyze the powder XRD (PXRD) pattern of the drop-casted colloids of MMHPbBr_3_. It is notoriously difficult to identify the crystal structure of lead halide perovskite NCs based on the laboratory PXRD, and more sophisticated techniques, like total scattering analysis, are often employed.^23^ A similar problem is observed for MMHPbBr_3_, for which the RT and HT phases have very similar simulated PXRD patterns. The small differences between the two are typically hard to resolve in the broadened peaks of the PXRD pattern of the NCs. A general comparison of the diffraction peak positions indicates that MMHPbBr_3_ NCs adopt the RT monoclinic structure. However, the observed shift of the diffraction peaks toward larger 2θ values in the experimental pattern when compared to the cubic modification can also be the consequence of the lattice expansion in the NCs compared to bulk. A more insightful picture can be gained from the total scattering and pair distribution function analysis (Figure 4h). Overall, MMHPbBr_3_ NC diffraction is better fitted with the RT monoclinic phase (χ^2^ = 12.23), but the fit to the HT cubic phase is not far behind (χ^2^ = 16.23). Since both RT and HT phases of MMHPbBr_3_ differ greatly in their local structure, we compare ^207^Pb solid-state NMR of the MMHPbBr_3_ bulk and NCs samples, which provides local information about the Pb environments (Figure 4i). Both bulk and NC spectra feature two peaks, one narrower centered around 400 ppm and the other broad spanning from 1000 to −1000 ppm. The narrower peak can be attributed to Pb in the nondistorted bromide octahedral environment, similar to other 3D perovskites, which show peaks broadened by *J*-coupling to the surrounding halides.^24^ The broader peak is attributed to the second lead species in the distorted, mixed halide-hydrazinium coordination. Commonly, longer Pb–X bonds, as well as increased coordination number, cause the ^207^Pb chemical shift to move upfield.^25^ The distorted coordination of PbBr_6_(MMH)_2_ features on average longer Pb–Br bonds compared to undistorted PbBr_6_ coordination (2.98 Å vs 3.10 Å, Figure S3), a consequence of two MMH^+^ coordinating to Pb. The increased broadening of the ^207^Pb peak correlates with the decreased symmetry of the coordination environment, leading to chemical shift anisotropy, additionally supporting the assignment. The features observed for NCs are very similar to those of the bulk material, with the main difference stemming from an additional broadening, commonly found for LHP NCs. The apparent change in relative intensity between the two peaks in the NC and bulk form can stem from the unequal broadening of the signals in the NC form. Another possible reason is that the relaxation times (T_1_ and T_2_) of the two signals can strongly depend on the morphology. Given the similar measurement conditions for both morphologies, one of the signals might have been attenuated more strongly compared to the other. Qualitatively, it is important to observe the presence of both peaks in bulk and nanomorphologies.

**Figure 5:**
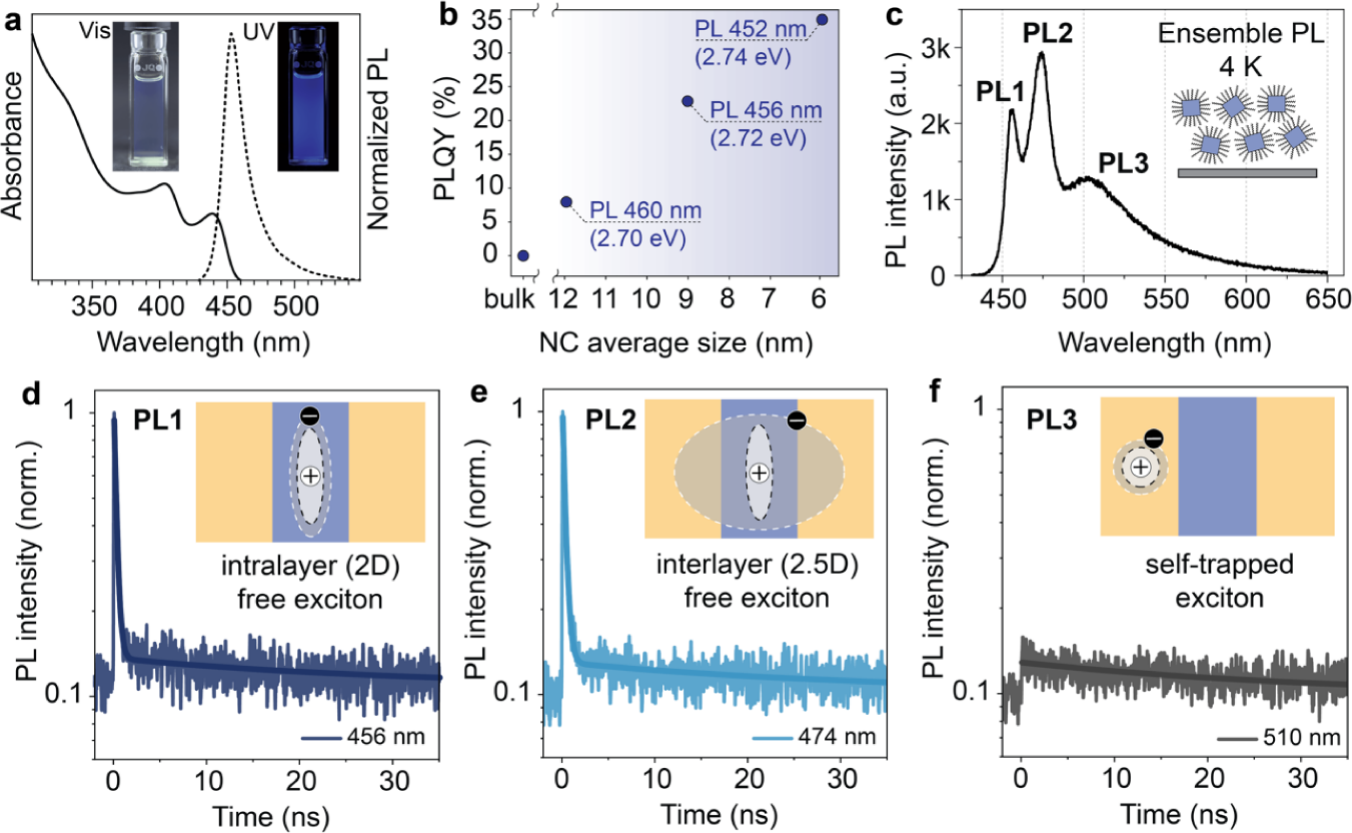
(a) PL and UV–vis spectra of an MMHPbBr_3_ NC colloid in *n*-hexane. (b) RT PL QY and PL peak wavelength of MMHPbBr_3_ colloids of various average NC sizes (see Figure S4 for TEM images). (c) Ensemble PL spectrum of a spin-coated MMHPbBr_3_ NC thin film at 4 K upon excitation at 405 nm, demonstrating three distinct PL bands. (d–f) PL decay traces measured at the maximum wavelength of the bands PL1 (d), PL2 (e), and PL3 (f), respectively. The insets depict the suggested excitonic nature for each PL band, with blue and gold indicating the undistorted and distorted octahedral layer and the dashed and solid lines sketching the presumed confinement potential wells, respectively.

To assess the exciton dynamics of the MMHPbBr_3_ NCs, we start with characterization at the NC-ensemble level. Figure 5a displays typical UV–vis absorption and steady-state PL spectra of MMHPbBr_3_ NCs at RT. The absorption spectrum features two distinct peaks at 445 and 405 nm, both present in the PL excitation (PLE) spectra (Figure S9). The PL exhibits a small Stokes shift, suggesting a small reorganization upon photoexcitation and the free character of the exciton. Consistent with the quantum size effect and a 3D delocalization of the electronic wave function, the MMHPbBr_3_ band gap increases with decreasing NC size (see Figure 4b). Concomitantly, decreasing the NC size also increases the PL QY at RT, from a negligible QY in bulk to about 35% for 6 nm NCs. Such size-dependent band gap and PL QY trends are typical for a 3D semiconductor. If we compare the sizing curves for similar-sized FAPbBr_3_ and CsPbBr_3_ NCs, the PL peak tunability from about 6 to 12 nm is 131 meV for FAPbBr_3_, 98 meV for CsPbBr_3_, and 48 meV for MMHPbBr_3_ (Table S2). Quantum confinement evidently plays a far smaller role in MMHPbBr_3_ NCs, although excitonic properties are still sensitive to the NC sizes. This serves as the first evidence that MMHPbBr_3_ is part of a new category of LHP semiconductors placed between 3D and 2D materials. Hence, we refer to MMHPbBr_3_ as a 2.5D semiconductor.

Upon cooling the MMHPbBr_3_ NC ensemble to cryogenic temperatures, several PL bands emerge (Figure S10) and at 4 K, we observe 3 distinct PL bands (PL1–PL3, Figure 5c) with 405 nm laser excitation. The two high-energy bands, PL1 (456 nm) and PL2 (474 nm), are both narrow, with a small Stokes shift (Figure S11) and short decay times in the subnanosecond range (Figures 5d,e and S12–S14), all pointing to the free excitonic nature of these PL bands. We hypothesize that the PL1 band originates from the intralayer free exciton (Figure 5d, inset), where both the electron and hole are localized in the periodic potential of 2D undistorted layers of MMHPbBr_3_. Conversely, a second free exciton band, PL2, can be associated with a free exciton, where one of the carriers (hole) is confined in a 2D undistorted layer, while the other carrier (electron) is delocalized over the whole 3D structure (Figure 5e, inset). This can also explain the higher-energy peak of PL1, in which the exciton is more confined with respect to PL2. In contrast to interlayer excitons in literature, where carriers are spatially separated and the PL decay time is significantly prolonged compared to intralayer excitons, the lifetime of the 2.5D exciton in this study is not very different from that of a 3D exciton (Figures S12 and S13). Compared to the two higher-energy PL bands, the third band, PL3, in the green spectral region (with a PL peak at around 510 nm), is significantly broader, features a large Stokes shift, and has a much longer lifetime (Figure 5f), all pointing toward a self-trapped exciton interpretation (Figure S11d) potentially arising from trapping in the distorted layer.^26^ We also note that a dual-emission band (at RT) was previously observed in 2D layered LHP systems, with interlayer or several-layer excitons as one of the possible explanations for the emergence of a red-shifted PL band.^27^

**Figure 6:**
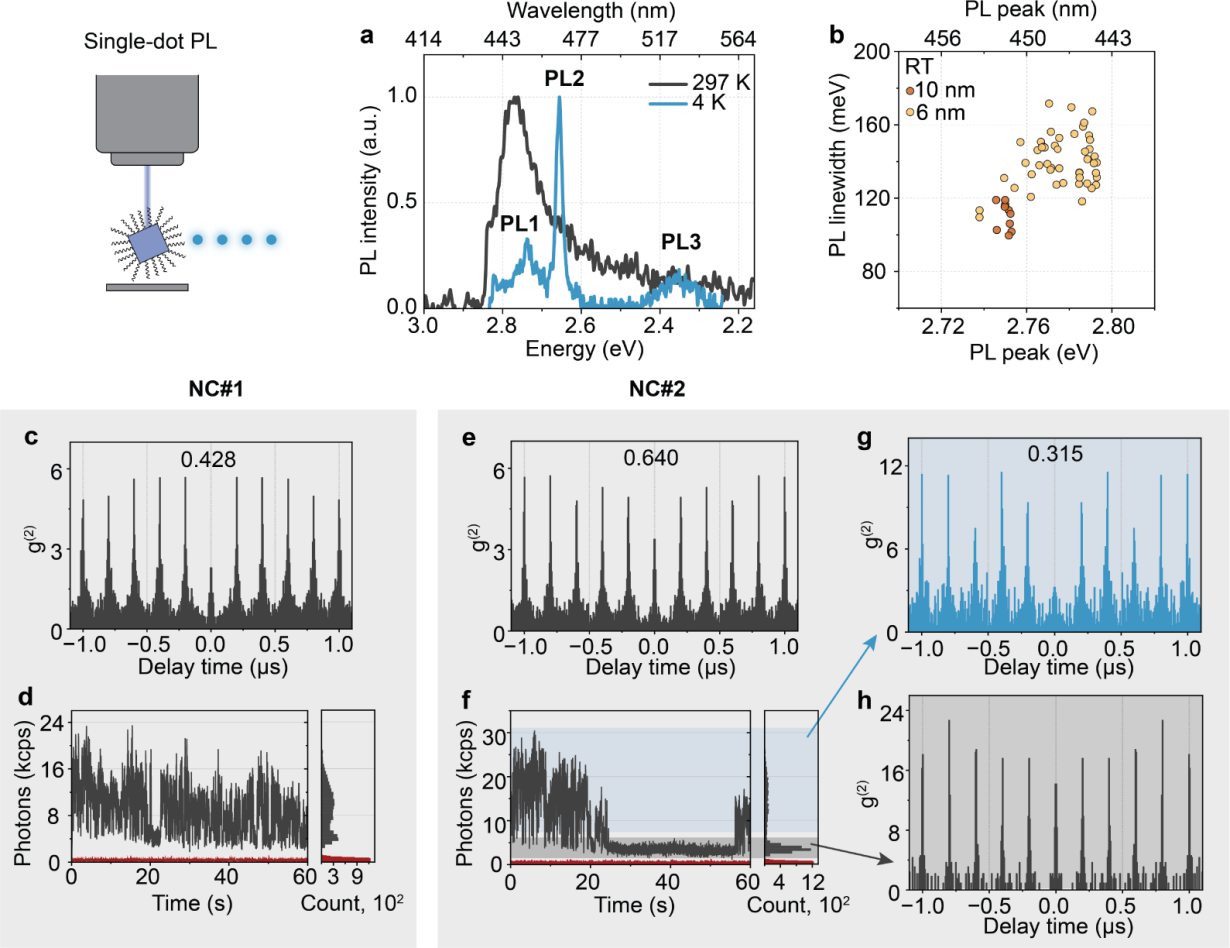
(a) PL spectra of a single MMHPbBr_3_ NC at 297 and 4 K. (b) Correlation of peak energy and line width of single MMHPbBr_3_ NC PL at 297 K, derived from NC colloids with an ensemble-averaged NC size of 6 nm (yellow markers) and 10 nm (orange markers), respectively. (c,e) Second-order intensity correlation (*g*^(2)^) and (d,f) PL intensity trace of two representative single NCs. Red traces are the background signal. (g,h) *g*^(2)^ extracted from (f) separately for bright and dim intensity levels defined via the blue and gray shaded areas, respectively.

Single-NC optical spectroscopy allows an intrinsic study of one emissive entity, avoiding the ensemble-averaging effect and complex interaction among NCs (Figure 6). Indeed, the steady-state PL spectrum of a single MMHPbBr_3_ NC features narrower bands than the ensemble PL (Figure 6a) due to the absence of ensemble inhomogeneities in the former. Interestingly, however, the single-NC PL spectrum qualitatively reproduces the features already observed at the ensemble level: one asymmetric band at RT and three bands at 4 K, with the latter comprising two narrow high-energy bands and one broad red-shifted band. The effects of quantum confinement were further demonstrated at the single-NC level, wherein both the emission energy and line width were systematically tuned by the NC size (Figure 6b). The observed increasing PL line width with decreasing NC size matches the trend commonly observed also in other size-confined nanostructures, which has previously been attributed to an increased softness of the NC surface, a stronger exciton wave-function overlap with the NC surface, as well as NC size confinement.^28^ For our MMHPbBr_3_ NCs, this trend is corroborated by the larger NCs (10 nm ensemble average size) showing significantly smaller PL line widths than the smaller NCs (6 nm ensemble average size). Furthermore, a narrower NC-to-NC distribution of the PL peak indicates weaker quantum confinement for the larger NCs.

2.5D electronic delocalization is unique in that the structure provides single-layer confinement, generating blue emission (2D-like), while simultaneously allowing the exciton wave function to occupy the NC volume by delocalizing across the periodic potential, thus exhibiting the quantum size effect (3D-like). For such an unprecedented system that has never been explored before, a pertinent question to ask is whether the 2.5D-confined NC behaves as a quantum emitter. The 2D analogue of the system is the *n* = 1 layered LHP, which emits classical light due to a continuum density of states, while 3D-confined LHP NCs are known to be excellent single-photon emitters. Therefore, the photon statistics of single MMHPbBr_3_ NCs were examined at room temperature. The emission from a single MMHPbBr_3_ NC exhibited antibunched photon statistics (Figure 6c), sharply distinguishing the 2.5D excitons from purely 2D-delocalized systems. Notably, the observed *g*^(2)^ values were relatively high (0.428 in Figure 6c) compared to similarly sized 3D-delocalized LHP NCs.^29^ One possibility is that the periodic potential provides a reservoir for spatially separated biexcitons, making them emissive by significantly lowering the chances of Auger recombination.^30^ Indeed, the PL intensity trace exhibited characteristic flickering and an above-background dim intensity level (Figure 6d). Another exemplary single NC is shown in Figure 6e,f, which is similar but with a *g*^(2)^ value above 0.5. It was confirmed that this signal originated from a single NC, rather than multiple NCs, by extracting the photon statistics separately for bright and dim intensity levels (Figure 6g,h). Bright bursts of neutral exciton emission followed single-photon statistics, while the less emissive dim state clearly featured multiphoton emission. The overall *g*^(2)^ value of 0.640 theoretically translates into about three individual emitters within a single NC. The fact that the periodic potential allows for the accommodation of multiple emissive two-level systems within a single particle is highly intriguing. Sharing context with moiré superlattices, 2.5D-semiconductor NCs herald the advent of a new class of materials for studying strongly correlated quantum phenomena and discovering unprecedented light-matter interactions.

## Conclusions

In conclusion, we present colloidal MMHPbBr_3_ NCs as a unique addition to the LHP family. Venturing into a rather unexplored field between typical 2D and 3D semiconductors, these emissive compounds host 2.5D excitons despite being formed by a 3D network of corner-sharing lead-halide octahedra. We rationalize this partial loss of dimensionality from a physical structure to an electronic structure by combining advanced structural characterization via X-ray diffraction, electron microscopy, and ^207^Pb solid-state NMR, with atomistic theory, using DFT calculations. First, the experimental characterization reveals alternating layers of distorted and undistorted lead-halide octahedra, consistent with the large size of the MMH A-site cations and their tendency for lone-pair expression. Second, DFT calculations rationalize the reduced electronic dimensionality by revealing 2D-delocalized holes and 3D-delocalized electrons. Leveraging the enhanced emissive character of our colloidal NCs, with a boost of the PL QY from essentially nonemissive bulk to 6 nm MMHPbBr_3_ NCs with 35% RT PL QY, we study the fundamental photophysical properties of such 2.5D excitons.

Being intermediate between 2D and 3D LHPs, MMHPbBr_3_ NCs feature only moderate quantum-size effects, with a PL blue shift of 48 meV when decreasing the NC size from 12 to 6 nm; such an increase of the band gap is less pronounced than in 3D LHPs (98 and 131 meV for similarly sized CsPbBr_3_ and FAPbBr_3_ NCs, respectively) and is thus consistent with the partial confinement of the exciton. Low-temperature PL spectroscopy at the ensemble and single-NC level reveals several emission bands at 4 K (PL1 at 456 nm, PL2 at 471 nm, and PL3 at 510 nm) that we tentatively attribute to free and trapped excitons with various confinements. Further experiments at the single-NC level revealed that the unique 2.5D electronic structure results in multiple emissive two-level systems residing within a single NC, contrary to pure single-photon emission observed for other 3D LHP NCs. We anticipate that this study will stimulate efforts in the unconventional structural design of periodic LHP structures and interest in nanostructuring novel inorganic materials to unlock nontrivial excitonic features.

## Supporting Information Available

Experimental methods (NC synthesis details) and characterization; ELF functions for various LHP cations; diffuse reflectance spectra of bulk MMHPbBr_3_; bonds and angles in the MMHPbB_3_ crystal structure; MMHPbBr_3_ NC analysis from TEM and 4D-STEM; ^31^P NMR of the surface-bound capping ligand; details of PDF analysis; size-dependent PL of MMHPbBr_3_ NCs; absorption and PLE of MMHPbBr_3_ NC colloids; temperature-dependent optical properties (PL, PLE, and TRPL) (PDF)
