# Supplementary material for: Highly Emissive Colloidal Nanocrystals of a “2.5-Dimensional” Monomethylhydrazinium Lead Bromide
Source: J Am Chem Soc. 2025 Feb 12;147(8):6795–804. doi: 10.1021/jacs.4c16698 (PMC11869272; doi:10.1021/jacs.4c16698)
Supplement: Supplementary file 1 — ja4c16698_si_001.pdf [file ja4c16698_si_001.pdf]

Supporting Information for:

Highly Emissive Colloidal Nanocrystals of a "2.5-dimensional" Monomethylhydrazinium Lead Bromide

Viktoriia Morad,[a,b] Taehee Kim,[a,b] Sebastian Sabisch,[a,b] Simon C. Boehme,[a,b] Simone Delessert,[a,b] Nadine J. Schrenker,[c] Sara Bals,[c] Gabriele Rainò,[a,b] Maksym V. Kovalenko[a,b*]

[a] Laboratory of Inorganic Chemistry, Department of Chemistry and Applied Biosciences, ETH Zürich, 8093 Zürich, Switzerland
[b] Empa – Swiss Federal Laboratories for Materials Science and Technology, Laboratory for Thin Films and Photovoltaics, 8600 Dübendorf, Switzerland
[c] Electron Microscopy for Materials Science (EMAT) and NANOlab Center of Excellence, University of Antwerp, 2020 Antwerp, Belgium

Content

Experimental methods (NC synthesis details) and characterization; ELF functions for various LHP cations; diffuse reflectance spectra of bulk $MMHPbBr_3$; bonds and angles in $MMHPbB_3$ crystal structure; $MMHPbBr_3$ NCs analysis from TEM and 4D-STEM; ^{31}P NMR of the surface-bound capping ligand; details of the PDF analysis; size-dependent PL of $MMHPbBr_3$ NCs; absorption and PLE of $MMHPbBr_3$ NCs colloid; temperature-dependent optical properties (PL, PLE, TRPL).

1. Materials and methods

DFT calculations. Ground state electronic structure of bulk $MMHPbBr_3$ was calculated with DFT using Vienna ab-initio simulation package (VASP) code.[1] The projector augmented wave (PAW) potentials for atoms were used. For the generalized gradient approximation (GGA),[2] the Perdew-Burke-Ernzerhof exchange-correlation functional (PBE) was used.[3] The kinetic energy cutoff of the plane-wave basis was set to 350 eV. A primitive reduced unit cell was used for calculations. Band structure calculations were performed along the high-symmetry K-points path generated with a density of 20 points. High-symmetry K-point path, reduced primitive unit cell, and band structure plot were obtained using sumo toolkit.[4] To analyze the spatial projections of electron and hole wavefunctions, partial charge densities (PARCHARG files), band and k-point decomposed, were calculated as implemented in VASP code using LPARD tag. The resulting partial charge densities in **Fig. 2** were plotted using Crystal Maker software.

Materials. All chemicals were used as purchased, without further purification. Methylhydrazine, lead (II) bromide (99.999%), cesium carbonate (Cs_2CO_3, 99.9%), hexane (≥99%), diisooctylphosphinic acid (DOPA, 90%), oleic acid (OA, 90%) were purchased from Sigma-Aldrich. Trioctylphosphine oxide (TOPO, 90%) and oleylamine (95%) were purchased from Strem Chemicals. Hydrobromic acid (HBr, 48% in water) was purchased from Acros.

$MMHPbBr_3$ NCs synthesis. The room temperature synthetic procedure was adopted from Ref.5. $PbBr_2$-TOPO stock solution was prepared by dissolving $PbBr_2$ (1 molar equivalent, *e.g.* 1 mmol) with TOPO (5 molar equivalents, *e.g.* 5 mmol) in n-octane (5 ml) initially at 120°C. When all $PbBr_2$ was dissolved, 20 ml n-hexane was added to obtain 0.04M concentration (density of TOPO taken for calculation is 0.88 g/cm^3). MMH stock solution was prepared by mixing MMH (32 uL) with DOPA (3 ml) and OA (2 ml) in n-octane (5 ml) at room temperature, resulting in 0.06M concentration. We note that n-octane is used either in the steps where heating is required (TOPO-$PbBr_2$) or where we have stored the precursor for a longer time (couple weeks) and gradual evaporation of more volatile solvent might have occurred and altered the concentration. Ligand (OAm or 2-octyl-1-dodecyl PEA) stock solutions with a concentration of 0.1 mg/uL were prepared in mesitylene because ligands are more polar and do not dissolve in alkanes. Choosing mesitylene over toluene also reduces the chance of concentration change over time due to solvent evaporation.

To obtain NCs, $PbBr_2$-TOPO aliquot was diluted with n-hexane and MMH stock precursor was swiftly injected. Immediately, a desired amount of ligand in mesitylene (0.1 mg/uL stock solution) was added to stop the reaction. For specific quantities of precursors, refer to **Table S1**. The NCs were purified by precipitation with 2-3 equivalents of antisolvent mixture (2:1 EtOAc:ACN, v:v), centrifugation at maximum speed for 30-60 seconds, and redispersing the pellet in n-hexane. Oleylammonium bromide (OAmBr) powder was prepared by reacting equimolar quantities of oleylamine and HBr water solution in absolute ethanol, by adding dropwise HBr to the cooled solution of oleylamine in ethanol. Precipiate (OAmBr) was collected by filtration and washed several times with diethylether. The synthesis of PEA ligand is reported elsewhere.[6]

Table S1. Quantities of precursors used for $MMHPbBr_3$ NCs synthesis with various sizes. The ligands that were used to cap NCs was either 2-octyl-1-dodecyl PEA or oleylammonium bromide (OAmBr).

NCs size range, nm	n-hexane, uL	$PbBr_2$-TOPO, uL	MMH, uL	Reaction time (s)	Ligand, (PEA) mg	Ligand (OAmBr), mg
10-12	1000	500	100	4	10	2.5
8-10	1000	500	100	1	10	2.5
6	3000	500	100	1	10	2.5

2. Characterization

4D-STEM characterization and HAADF imaging were performed with a probe-corrected Thermo Fisher Titan Themis at an acceleration voltage of 200 kV and a semi-convergence angle of the electron beam of 13.5 mrad. The 4D-STEM datasets are acquired with a custom-made Timepix3 detector,[7] which is an event-driven hybrid pixelated direct electron detector. The 4D-STEM datasets were reconstructed with a recently developed neural network, see Ref.[8] for a detailed description.

Solid-state ^{207}Pb NMR for bulk and NC powder of $MMHPbBr_3$ was measured at 14.1 T using 3.2 mm double resonance magic angle spinning probe and a Bruker Avance III HD spectrometer. To avoid sintering of the NCs during the measurements both spectra were recorded under static conditions at room temperature. Due to the broad nature of the lines both spectra were recorded using three-pulse excitation with a pulse length of 4.5 us. The delay between pulses was set to 5 us while the recycle delay was set to 2s for the bulk and 1s for the NCs. In total 128 thousand traces were recorded for both samples.

Total scattering and pair distribution function analysis. Shortly before the total scattering measurement the colloidal NC sample (average size 8.5 ± 1.1 nm as determined from TEM, Fig. S4j) was dried and transferred to a 0.3mm glass capillary, which was sealed before measurement. For the X-Ray Powder Diffraction total scattering measurements, a STADI P from STOE, equipped with an Ag Kα1-radiation source and 4 modules Mythen2 1K silicon strip detectors, was used. Each measurement was carried out for 22h divided into 8 ranges resulting in a 2ϑ range from - 8.5 to 145.07. The ranges were summed up using WinXPow, resulting in a slightly reduced 2ϑ range. Using xPDFsuite a background correction, using a background measurement of either an empty or a native solvent-filled capillary was carried out. Qmin was determined by the instrumental limitations given by the zero-beam stopper used. Qmax was determined by evaluating the S/N ratio of the background corrected data, by eye. xPDFsuite was used for background correction and generating the PDFs from the total scattering XRDs,. Additionally, to determine occupancies as well as sizes the integrated software package PDFgui with a models built on the cif files from Ref. 9 was used.

Steady-state ensemble PL from a colloid of $MMHPbBr_3$ NCs (**Fig. 4a**) was recorded with FluoroMax4-Plus-P (Horiba Jobin Yvon), equipped with 150 W Xenon lamp.

UV-Vis spectra were recorded with a Jasco V670 spectrophotometer equipped with a deuterium (D2) lamp (190 –350 nm) for use in UV, a halogen lamp (330 –2700 nm) for use in UV/NIR, and an integrating sphere (ILN-725) with a working wavelength range of 220 –2200 nm. For solids (**Fig. S2**), the absorbance spectrum was estimated from diffuse reflectance measured on the powdered crystals transformed into Kubelka-Munk function.

Absolute quantum yields were measured using a Quantaurus-QY Plus spectrometer from Hamamatsu.

Ensemble PL at 4K and TR PL traces (**Fig. 4b-f**). NC solution (~1 mg/ml) was drop-casted onto a crystalline Si wafer with a thermal oxide layer of 2 μm thickness. This sample was mounted inside an evacuated cryostat with closed-loop liquid helium system which was cooled down to a target temperature of 4 K. NC ensemble film was excited by the fiber-coupled pulsed laser with the repetition rate of 10 MHz, at 405 nm. Excitation power density was 1.4 nJ/cm^2. PL spectra were recorded with an EMCCD coupled to a monochromator (Princeton Instruments). Time-resolved PL traces were recorded by using an APD (MPD, irf = 50 ps) placed at the exit port of the monochromator, accepting the photons only at the selected wavelength.

Single-NC spectroscopy. Sample was prepared by diluting the NC colloid solution (~1 mg/ml) by a factor of 400 in n-octane (Sigma-Aldrich, anhydrous, ≥99%), then diluting once more by factor of 30 in a 1-mass% solution of styrene-ethylene-butylene-styrene (SEBS) polymer in cyclohexane (Sigma-Aldrich, anhydrous, 99.5%). 40 μl of final solution was spin-coated onto a glass coverslip (for RT measurement) and onto an intrinsic crystalline Si wafer with a thermal oxide layer of 2 μm thickness (for cryo-T measurement). For single-NC spectroscopy, a custom-built μ-PL setup was used. Samples were mounted on XYZ nano-positioning stages (Smaract for RT), (Attocube for cryo-T) inside a cryostat which was evacuated and cooled down to a target temperature of 4 K with closed-loop liquid helium system. Single NCs were excited using a diode laser at 405 nm (LDH-D-C-405, PicoQuant), coupled to the optical fiber. Laser beam was focused on the sample by a microscope objective (oil-immersive, NA 1.3, 100x for RT; air, NA 0.8, 100x for cryo-T) to excite the single NCs. Power densities used to excite single NCs were 25 μJ/cm^2 for RT and 0.03-0.13 μJ/cm^2 for cryo-T. NC emission was collected by the identical objective lens and passed through the long-pass filter with cut-off wavelength at 430 nm. A monochromator coupled with an EMCCD (Princeton Instruments) was used to record the spectra. Photon statistics were collected using a HBT setup consisting of two APDs, 50/50 beam splitter, and a TCSPC module (PicoQuant).

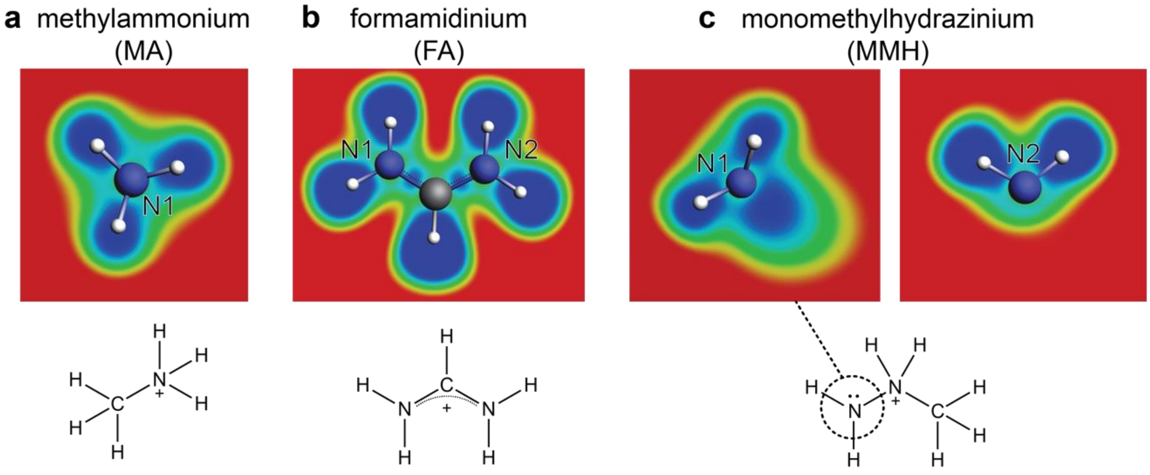

Figure S1. Electron localization functions (ELF) calculated for FA, MA and MMH, projected on a 2D plane to showcase the lone pair on one of the nitrogen atoms of MMH. ELFs were calculated and visualized as implemented with Amsterdam Density Functional package[10] with LDA functional and DZ basis set.

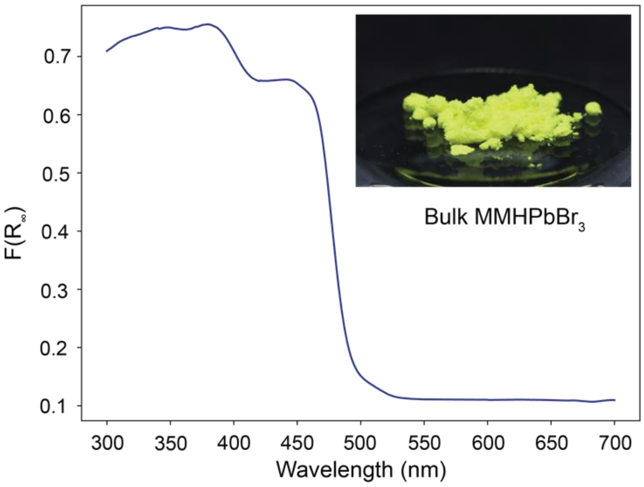

Figure S2. Kubelka-Munk function ($F(R_\infty)=(1-R_\infty)^2/2R_\infty$, where R_∞ is diffuse reflectance) of the bulk powder of $MMHPbBr_3$.

a

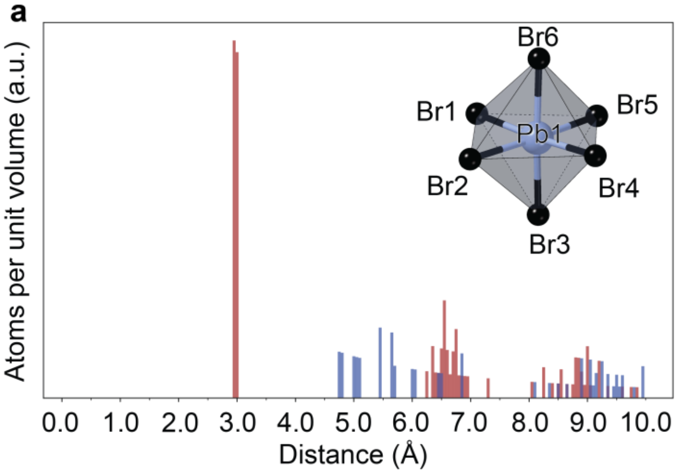

Bond	From	To	d [Å]
Pb - Br	Pb1	Br1	2.95464
Pb - Br	Pb1	Br3	2.95937
Pb - Br	Pb1	Br2	2.97933
Pb - Br	Pb1	Br4	3.00115
Pb - Br	Pb1	Br5	3.00473
Pb - Br	Pb1	Br6	3.01132

b

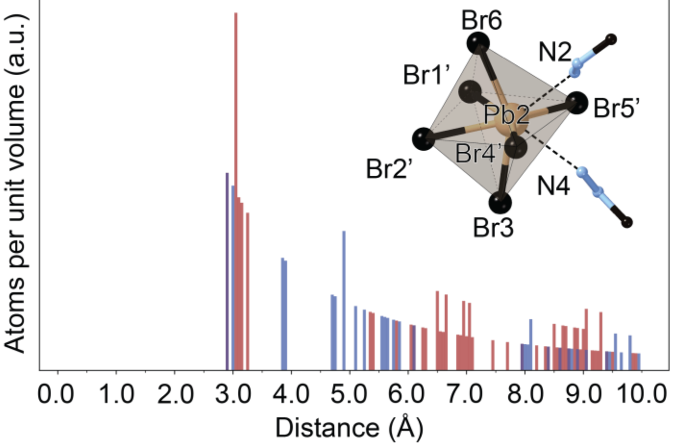

Bond	From	To	d [Å]
Pb - N	Pb2	N2	2.91532
Pb - Br	Pb2	Br4’	2.94051
Pb - N	Pb2	N4	3.04444
Pb - Br	Pb2	Br3	3.05279
Pb - Br	Pb2	Br6	3.06236
Pb - Br	Pb2	Br2’	3.10119
Pb - Br	Pb2	Br5’	3.16585
Pb - Br	Pb2	Br1’	3.27578

c

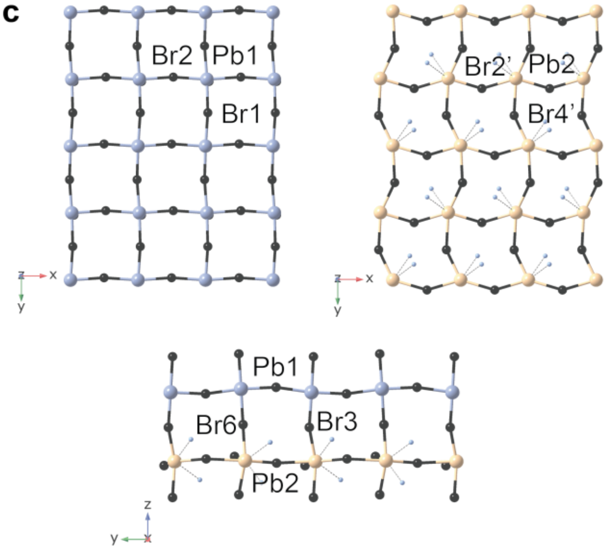

Angle	(°)
Pb1 - Br2 - Pb1	172.17
Pb1 - Br1 - Pb1	167.87
Pb1 - Br3 - Pb2	156.69
Pb1 - Br6 - Pb2	170.29
Pb2 - Br2’ - Pb2	144.58
Pb2 - Br4’ - Pb2	150.79

Figure S3. Crystal structure details of $MMHPbBr_3$. (**a-b**) Pb-X (Br, N) distances for undistorted (a) and distorted (b) Pb coordinations. (**c**) Various Pb-Br-Pb angles in $MMHPbBr_3$.

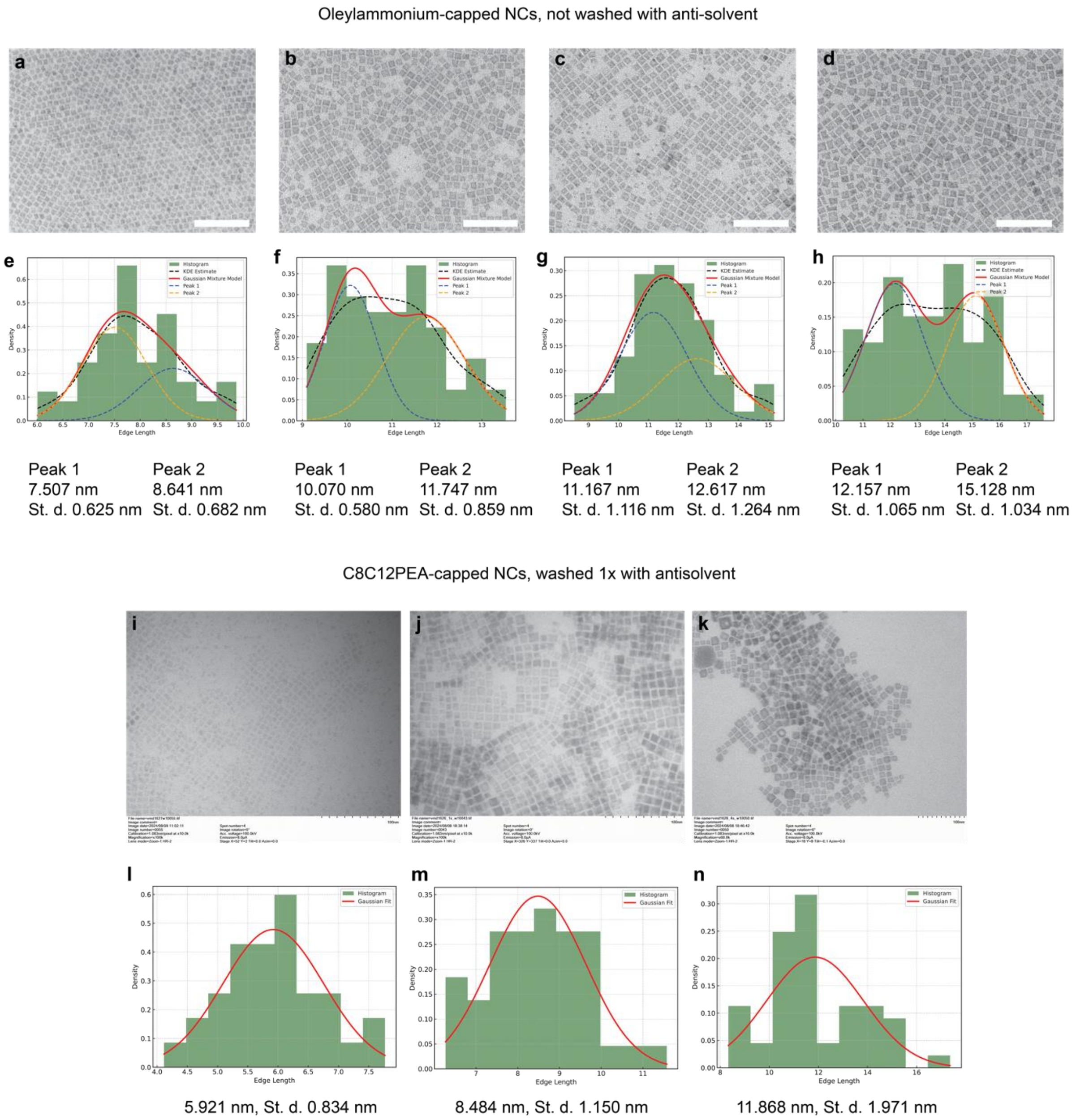

Figure S4. (**a-d**) TEM images of $MMHPbBr_3$ NCs capped with oleylammonium, not purified. (**e-h**) Estimation of average NCs sizes in the sample by fitting with two Gaussian peaks, because of non-cuboidal shape of the $MMHPbBr_3$ NCs. (**i-k**) TEM images of $MMHPbBr_3$ NCs capped with 2-octy-1-dodecyl PEA ligand, synthesized with precursor quantities from **Table S1**, representing samples of various average sizes: 6 nm (**i**), 8-10 nm (**j**) and 10-14 nm (**k**). NCs were purified once with anti-solvent (EtOAc:ACN 2:1) precipitation and solvent (hexane) redispersion and stored under ambient conditions. (**l-n**), Distribution of NC edges measured from images (**i-k**) and fitted with Gaussian model to obtain average sizes, for NCs count 50 in each case.

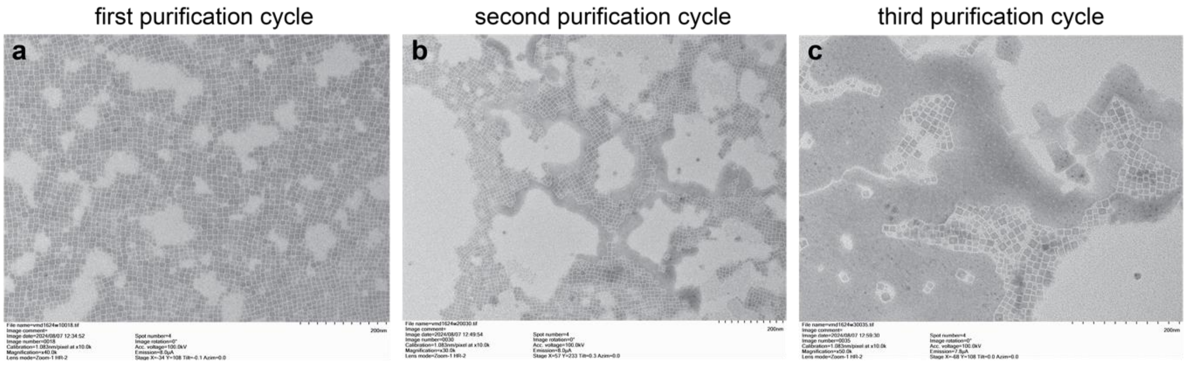

Figure S5. (**a-c**) TEM images of the 2-octyl-1-dodecyl PEA capped $MHAPbBr_3$ NCs purified with anti-solvent precipitation once (**a**), twice (**b**) and thrice (**c**) showing signs of NCs degradation.

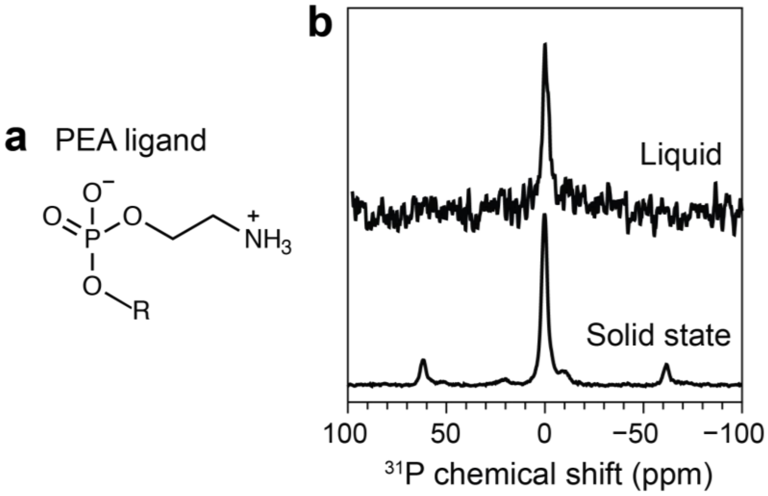

Figure S6. (**a**) Chemical formula of the phosphoethanolammonium (PEA) zwitterionic ligand used to stabilize colloids of $MMHPbBr_3$ (R = 2-octy-1-dodecyl). (**b**) ^{31}P liquid and solid-state NMR of the antisolvent-purified $MMHPbBr_3$ NCs colloid and dried NCs powder, respectively, attesting to phosphate group binding to surface Pb similarly to other lead bromide perovskites.[6]

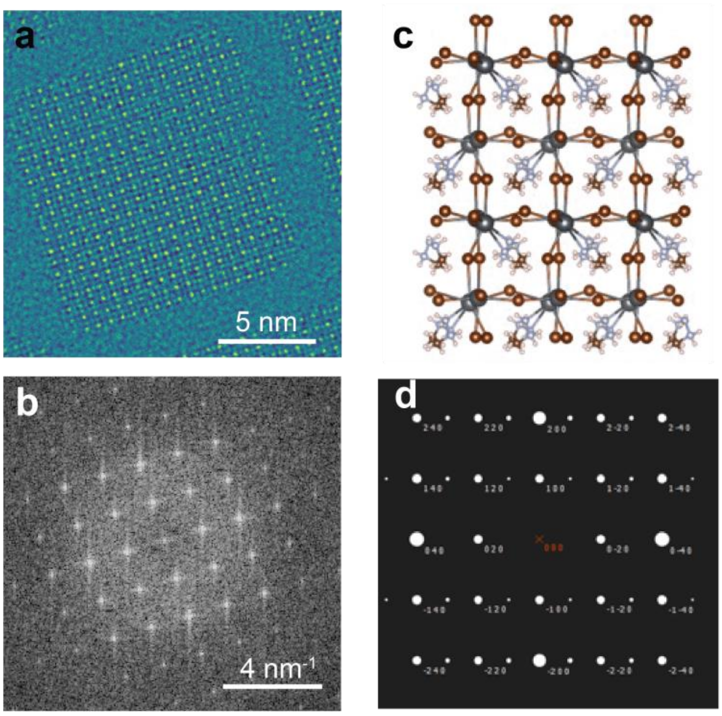

Figure S7. 4D-STEM phase contrast reconstruction of $MMHPbBr_3$ NC. (**a-b**) 4D-STEM phase contrast reconstruction of an $MMHPbBr_3$ NC (**a**) and the corresponding FFT (**b**). (**c-d**) Experimental FFT agrees with the [001] zone axis of $MMHPbBr_3$ (**c**) and corresponding theoretical FFT (**d**).

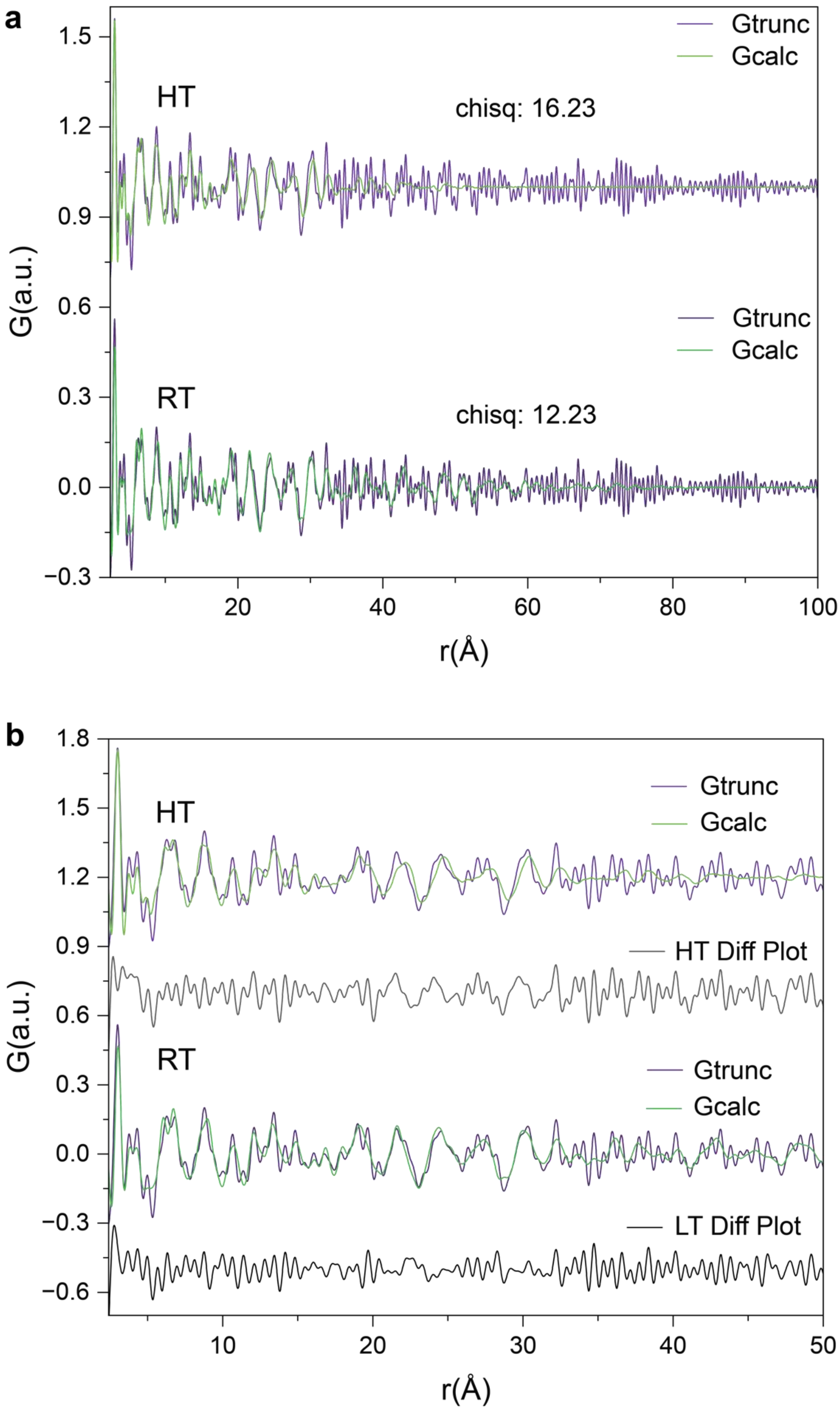

Figure S8. (**a**) PDF analysis of the XRD data of $MMHPbBr_3$ NCs. The fits to calculated models based on RT (monoclinic) and HT (cubic) phases of $MMHPbBr_3$. RT model has a better fit with χ^2 of 12.23 over 16.23 for the HT model. The corresponding Rw values are 0.653 for the HT phase model and 0.567 for the RT phase model. The average

size of the ensemble determined from RT model is 9.57 nm, which is in a good agreement with average size determined from TEM (8.5±1.1 nm). (**b**) The difference plot for the experimental and model for HT and LT phases.

Table S2. Size-dependent PL of $CsPbBr_3$[11], $FAPbBr_3$[12,13] and $MMHPbBr_3$ (this work).

Composition	Size (nm)	PL peak (eV)	PL tunability/
$CsPbBr_3$	12.8	2.398	131
	6.2	2.496	
$FAPbBr_3$	12	2.339	98
	6.3	2.470	
$MMHPbBr_3$	12	2.695	48
	6	2.743	

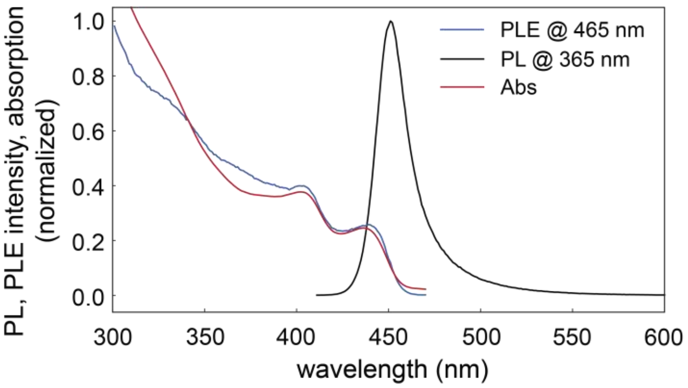

Figure S9. Ensemble PL, absorption, and PLE for an $MMHPbBr_3$ NCs colloid in n-hexane, displaying agreement between absorption and PLE spectra.

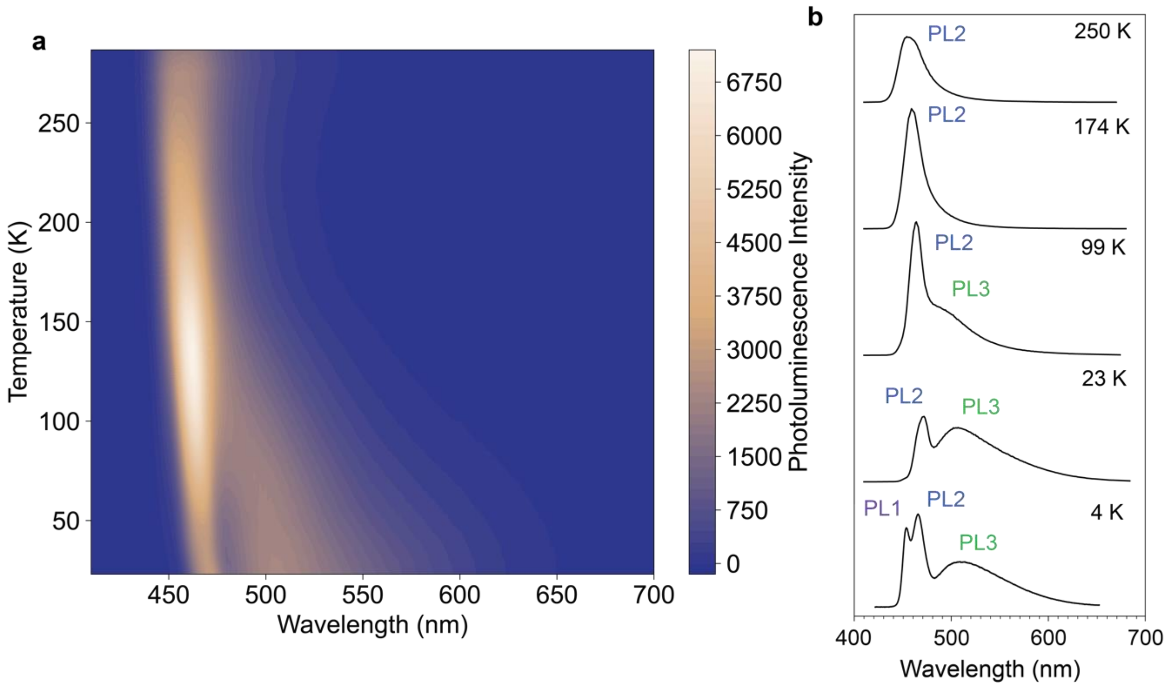

Figure S10. (**a**) Map of the temperature dependence of PL for a spin-coated MMHPbBr$_3$ NCs film. (**b**) Corresponding 1D slices at different temperatures show the evolution of three PL peaks.

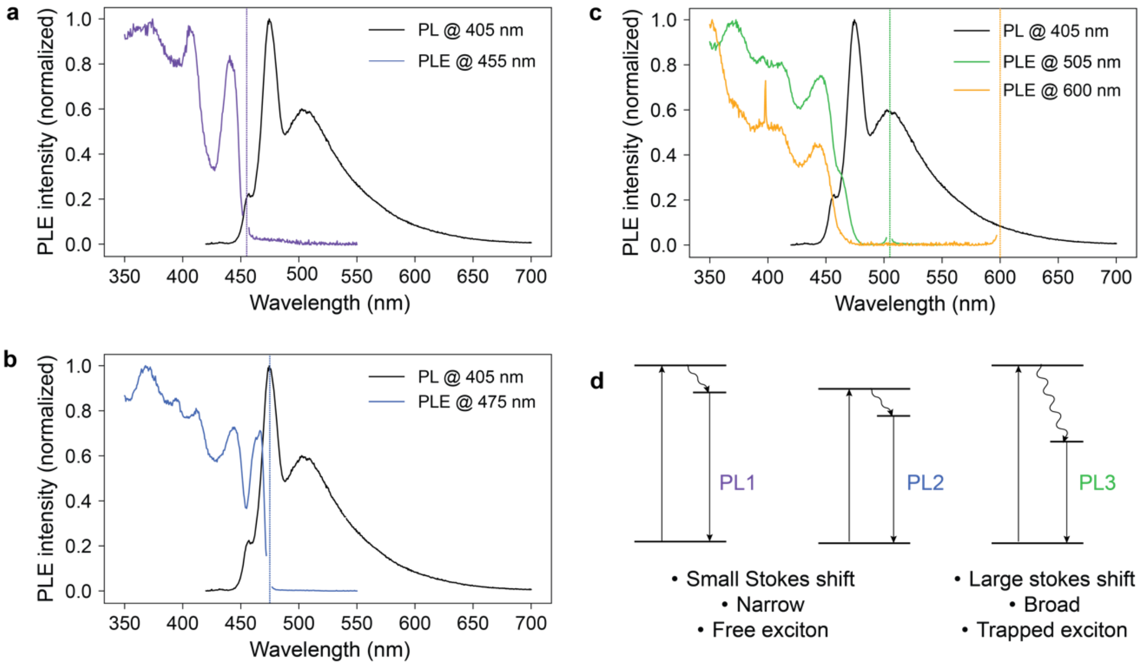

Figure S11. (**a-c**) PL and PLE spectra of MMHPbBr$_3$ NCs ensemble (spin-coated thin film) at 17K. (**d**) Schematics of the energetic alignment of electronic states involved in PL1, PL2 and PL3 emission processes.

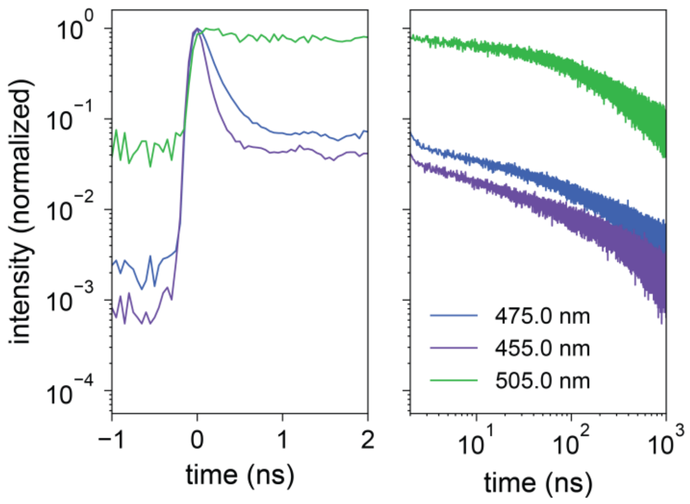

Figure S12. Exponential decays of the three PL bands measured at 17K.

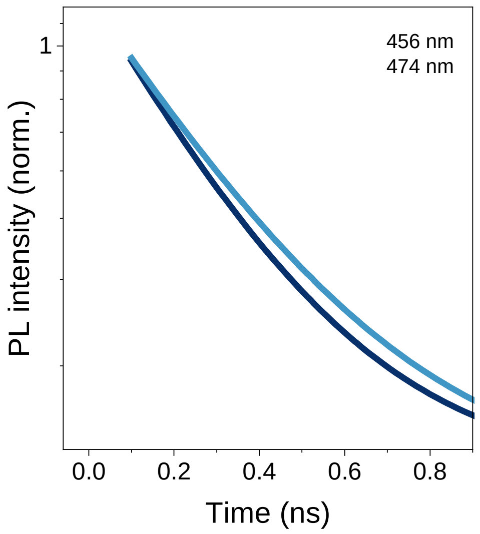

Figure S13. Exponential decay fit of PL1 (456 nm) and PL2 (474 nm) TR PL traces @4K.

Supplementary References

(1) Kresse, G.; Hafner, J. Ab Initio Molecular Dynamics for Liquid Metals. *Phys. Rev. B* **1993**, *47* (1), 558–561. https://doi.org/10.1103/PhysRevB.47.558.
(2) Perdew, J. P.; Burke, K.; Ernzerhof, M. Generalized Gradient Approximation Made Simple. *Phys. Rev. Lett.* **1996**, *77* (18), 3865–3868. https://doi.org/10.1103/PhysRevLett.77.3865.
(3) Blöchl, P. E. Projector Augmented-Wave Method. *Phys. Rev. B* **1994**, *50* (24), 17953–17979. https://doi.org/10.1103/PhysRevB.50.17953.
(4) Ganose, A. M.; Jackson, A. J.; Scanlon, D. O. Sumo: Command-Line Tools for Plotting and Analysis of Periodic *ab Initio* Calculations. *Journal of Open Source Software* **2018**, *3* (28), 717. https://doi.org/10.21105/joss.00717.
(5) Akkerman, Q. A.; Nguyen, T. P. T.; Boehme, S. C.; Montanarella, F.; Dirin, D. N.; Wechsler, P.; Beiglböck, F.; Rainò, G.; Erni, R.; Katan, C.; Even, J.; Kovalenko, M. V. Controlling the Nucleation and Growth Kinetics of Lead Halide Perovskite Quantum Dots. *Science* **2022**, *377* (6613), 1406–1412. https://doi.org/10.1126/science.abq3616.
(6) Morad, V.; Stelmakh, A.; Svyrydenko, M.; Feld, L. G.; Boehme, S. C.; Aebli, M.; Affolter, J.; Kaul, C. J.; Schrenker, N. J.; Bals, S.; Sahin, Y.; Dirin, D. N.; Cherniukh, I.; Raino, G.; Baumketner, A.; Kovalenko, M. V. Designer Phospholipid Capping Ligands for Soft Metal Halide Nanocrystals. *Nature* **2024**, *626* (7999), 542–548. https://doi.org/10.1038/s41586-023-06932-6.
(7) Poikela, T.; Plosila, J.; Westerlund, T.; Campbell, M.; Gaspari, M. D.; Llopart, X.; Gromov, V.; Kluit, R.; Beuzekom, M. van; Zappon, F.; Zivkovic, V.; Brezina, C.; Desch, K.; Fu, Y.; Kruth, A. Timepix3: A 65K Channel Hybrid Pixel Readout Chip with Simultaneous ToA/ToT and Sparse Readout. *J. Inst.* **2014**, *9* (05), C05013. https://doi.org/10.1088/1748-0221/9/05/C05013.
(8) Friedrich, T.; Yu, C.-P.; Verbeeck, J.; Van Aert, S. Phase Object Reconstruction for 4D-STEM Using Deep Learning. *Microscopy and Microanalysis* **2023**, *29* (1), 395–407. https://doi.org/10.1093/micmic/ozac002.
(9) Mączka, M.; Ptak, M.; Gągor, A.; Stefańska, D.; Zaręba, J. K.; Sieradzki, A. Methylhydrazinium Lead Bromide: Noncentrosymmetric Three-Dimensional Perovskite with Exceptionally Large Framework Distortion and Green Photoluminescence. *Chem. Mater.* **2020**, *32* (4), 1667–1673. https://doi.org/10.1021/acs.chemmater.9b05273.
(10) te Velde, G.; Bickelhaupt, F. M.; Baerends, E. J.; Fonseca Guerra, C.; van Gisbergen, S. J. A.; Snijders, J. G.; Ziegler, T. Chemistry with ADF. *Journal of Computational Chemistry* **2001**, *22* (9), 931–967. https://doi.org/10.1002/jcc.1056.
(11) Brennan, M. C.; Herr, J. E.; Nguyen-Beck, T. S.; Zinna, J.; Draguta, S.; Rouvimov, S.; Parkhill, J.; Kuno, M. Origin of the Size-Dependent Stokes Shift in CsPbBr3 Perovskite Nanocrystals. *J. Am. Chem. Soc.* **2017**, *139* (35), 12201–12208. https://doi.org/10.1021/jacs.7b05683.
(12) Protesescu, L.; Yakunin, S.; Bodnarchuk, M. I.; Bertolotti, F.; Masciocchi, N.; Guagliardi, A.; Kovalenko, M. V. Monodisperse Formamidinium Lead Bromide Nanocrystals with Bright and Stable Green Photoluminescence. *J. Am. Chem. Soc.* **2016**, *138* (43), 14202–14205. https://doi.org/10.1021/jacs.6b08900.
(13) Li, Y.; Ding, T.; Luo, X.; Tian, Y.; Lu, X.; Wu, K. Synthesis and Spectroscopy of Monodispersed, Quantum-Confined FAPbBr3 Perovskite Nanocrystals. *Chem. Mater.* **2020**, *32* (1), 549–556. https://doi.org/10.1021/acs.chemmater.9b04297.
